# A Complex of Cas Proteins 5, 6, and 7 Is Required for the Biogenesis and Stability of Clustered Regularly Interspaced Short Palindromic Repeats (CRISPR)-derived RNAs (crRNAs) in Haloferax volcanii*[Fn FN2]

**DOI:** 10.1074/jbc.M113.508184

**Published:** 2014-01-23

**Authors:** Jutta Brendel, Britta Stoll, Sita J. Lange, Kundan Sharma, Christof Lenz, Aris-Edda Stachler, Lisa-Katharina Maier, Hagen Richter, Lisa Nickel, Ruth A. Schmitz, Lennart Randau, Thorsten Allers, Henning Urlaub, Rolf Backofen, Anita Marchfelder

**Affiliations:** From the ‡ Department of Biology II, Ulm University, 89069 Ulm, Germany,; the § Bioinformatics Group, Department of Computer Science, University of Freiburg, Georges-Köhler-Allee 106, 79110 Freiburg, Germany,; the ¶ Max Planck Institute for Biophysical Chemistry, Am Fassberg 11, 37077 Göttingen, Germany,; the ** Max Planck Institute for Terrestrial Microbiology, Karl-von-Frisch-Strasse 10, 35043 Marburg, Germany,; the ‡‡ Institute for General Microbiology, Christian-Albrechts-Universität Kiel, 24118 Kiel, Germany,; the §§ School of Life Sciences, University of Nottingham, Queen's Medical Centre, Nottingham NG7 2UH, United Kingdom,; the ¶¶ Centre for Biological Signalling Studies (BIOSS), Cluster of Excellence, University of Freiburg, 79110 Freiburg, Germany, and; ‖ Bioanalytics, Institute of Clinical Chemistry, University Medical Center Göttingen, Robert-Koch-Strasse 40, 37075 Göttingen, Germany

**Keywords:** Archaea, Microbiology, Molecular Biology, Molecular Genetics, Protein Complexes, CRISPR/Cas, Cas6, Haloferax volcanii, crRNA, Type I-B

## Abstract

The clustered regularly interspaced short palindromic repeats/CRISPR-associated (CRISPR-Cas) system is a prokaryotic defense mechanism against foreign genetic elements. A plethora of CRISPR-Cas versions exist, with more than 40 different Cas protein families and several different molecular approaches to fight the invading DNA. One of the key players in the system is the CRISPR-derived RNA (crRNA), which directs the invader-degrading Cas protein complex to the invader. The CRISPR-Cas types I and III use the Cas6 protein to generate mature crRNAs. Here, we show that the Cas6 protein is necessary for crRNA production but that additional Cas proteins that form a CRISPR-associated complex for antiviral defense (Cascade)-like complex are needed for crRNA stability in the CRISPR-Cas type I-B system in *Haloferax volcanii in vivo*. Deletion of the *cas6* gene results in the loss of mature crRNAs and interference. However, cells that have the complete *cas* gene cluster (*cas1–8b*) removed and are transformed with the *cas6* gene are not able to produce and stably maintain mature crRNAs. crRNA production and stability is rescued only if *cas5*, -*6*, and -*7* are present. Mutational analysis of the *cas6* gene reveals three amino acids (His-41, Gly-256, and Gly-258) that are essential for pre-crRNA cleavage, whereas the mutation of two amino acids (Ser-115 and Ser-224) leads to an increase of crRNA amounts. This is the first systematic *in vivo* analysis of Cas6 protein variants. In addition, we show that the *H. volcanii* I-B system contains a Cascade-like complex with a Cas7, Cas5, and Cas6 core that protects the crRNA.

## Teaser

**Background:** The Cas6 protein is required for generating crRNAs in CRISPR-Cas I and III systems.

**Results:** The Cas6 protein is necessary for crRNA production but not sufficient for crRNA maintenance in *Haloferax*.

**Conclusion:** A Cascade-like complex is required in the type I-B system for a stable crRNA population.

**Significance:** The CRISPR-Cas system I-B has a similar Cascade complex like types I-A and I-E.

## Introduction

Prokaryotes have developed a variety of resistance mechanisms to defend themselves against invaders. Recently, a new defense mechanism was detected: the CRISPR-Cas^2^ system (for reviews, see Refs. 1–6). Key elements of this defense system are the Cas proteins and the CRISPR RNA. The latter consists of short repeat sequences that are separated by variable sequences (spacers). Spacer sequences are derived from previous invaders; therefore, the CRISPR locus is a memory of all previous attacks by invaders.

The defense reaction is divided into three stages. In the adaptation stage, the invader DNA is cleaved, and a piece of it is selected to be integrated as a new spacer into the CRISPR locus, where it is stored as an identity tag for future attacks by this invader. During the second stage (the expression stage), the CRISPR RNA (pre-crRNA) is transcribed and subsequently processed into the mature crRNAs. In the third stage (the interference stage), Cas proteins, together with crRNAs, identify and degrade the invader.

The CRISPR-Cas systems have been sorted into three major classes (I–III) that are further subdivided into 11 subtypes (I-A to -F, II-A to -C, and III-A and -B) (7, 8). In CRISPR-Cas types I and III, the mature crRNA is generally generated by a member of the Cas6 protein family (9). This endonuclease has been analyzed in detail in several bacteria (10–14) and a few archaea (10–17). The Cas6 proteins contain a ferredoxin fold and a characteristic glycine-rich motif at the C terminus (8, 18, 19). All Cas6 proteins analyzed to date generate a crRNA with an 8-nucleotide-long 5′-handle (16, 20–23). The crRNAs generated by the Cas6 proteins usually contain a 2′-3′ phosphate group (14); only processing by the type I-F Cas6 (Cas6f) results in a non-cyclic phosphate group (24). The Cas6e and Cas6f proteins stay bound to the processed crRNA (21, 25, 26). Apart from these similarities, the various Cas6 proteins show several differences; they share very little sequence identity and show differences in catalytic site composition and in the molecular details of the reaction. The Cas6 protein from *Pyrococcus furiosus* contains a catalytic triad consisting of a tyrosine, a histidine, and a lysine (14), whereas the Cas6e protein from *Thermus thermophilus* (type I-E) contains a catalytic dyad consisting of a tyrosine and a histidine (25, 27). Studies with the Cas6b protein from *Methanococcus maripaludis* (type I-B) identified two histidine residues important for catalysis (16). The *Sulfolobus solfataricus* Cas6 protein does not contain a histidine close to the catalytic site but seems to require a network of basic residues for catalytic activity (28). Cas6e (20, 25, 27, 29) and Cas6f are known to bind to stable hairpin motifs on the repeat (21, 26). The Cas6 from *Staphylococcus epidermidis* binds to a smaller hairpin structure (30), whereas the Cas6 from *P. furiosus* binds to an unstructured repeat (10, 31, 32). Type I-C systems do not have a Cas6 protein because the Cas5d protein is responsible for crRNA processing (33, 34).

Whereas in system III, the Cas6 protein acts alone, in type I-A, I-E, and I-F systems, the respective Cas6 protein is part of the CRISPR-associated complex for antiviral defense (Cascade) (9). The Cascade complex is composed of different Cas proteins, depending on the subtype, and it is involved in pre-crRNA processing. The mature crRNA remains associated with the complex, which subsequently binds to the invader DNA. The invader is then degraded by the Cas3 protein (35, 36). The composition of the type I-B Cascade complex has not yet been described.

It has been suggested that the Cas6 proteins co-evolved with the CRISPR RNA repeat sequences (37, 38). Repeat sequences have been shown to have highly variable structures. Some form hairpin structures, whereas others do not, and they have been clustered into different groups (37, 39). Because the Cas6 proteins interact with the crRNAs, they have to adapt to their respective repeat sequences. Taken together, the current data about the Cas6 proteins show that these proteins are highly divergent from each other. To fully understand this class of proteins, more data about different Cas6 proteins from different subtypes and organisms are required.

**FIGURE 1.**
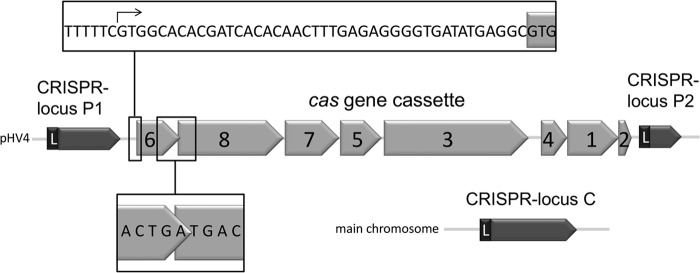
**Location of *H. volcanii cas* genes and CRISPR loci.** The *cas* gene cluster is located on the minichromosome pHV4 and encodes the Cas1, Cas2, Cas3, Cas4, Cas5, Cas6, Cas7, and Cas8b proteins, the latter being the signature protein for subtype I-B. The cluster is flanked by two CRISPR RNA loci (P1 and P2). CRISPR locus P1 contains 17 repeats and 16 spacers, and locus P2 contains 12 repeats and 11 spacers. The third CRISPR RNA locus is encoded on the main chromosome and contains 25 repeats and 24 spacers. The leader region (*L*) at the 5′-end of the CRISPR locus contains the promoter. The 5′-end of the *cas6* mRNA was mapped with 5′-RACE and is indicated in the *box above* the *cas* gene cluster by an *arrow*. The overlap of the *cas6* and *cas8b* genes is shown in the *box below* the *cas* gene cluster.

Here, we report new data about the Cas6 protein from *Haloferax volcanii. H. volcanii* is a halophilic archaeon that requires 2.1 m NaCl for optimal growth and contains similar concentrations of salt intracellularly to cope with the high salt concentration in the medium. *H. volcanii* is studied as an archaeal model organism because it is easy to cultivate and to genetically modify. *H. volcanii* encodes a CRISPR-Cas type I-B system with three different CRISPR loci: two located in close proximity to each other on the chromosomal plasmid pHV4 (locus P1 and P2) and one on the main chromosome (locus C) (Fig. 1) (40, 41). All three loci are expressed and processed (42). The *cas* gene cluster, which encodes the Cas proteins Cas1–8b, is located between the two plasmid-encoded CRISPR loci. The repeat sequences of the *H. volcanii* CRISPR RNA have the potential to form a short stem loop structure (23).

Here, we present the first systematic, *in vivo* mutational analysis of a Cas6 protein and its effects on the cellular crRNA amounts as well as the interference reaction. Furthermore, we show that the *H. volcanii* I-B system contains a Cascade-like complex consisting of at least Cas5, Cas6, and Cas7 proteins as well as crRNA.

## EXPERIMENTAL PROCEDURES

### 

#### 

##### Strains

*H. volcanii* strains H119Δ*cas6* (Δ*pyrE2*, Δ*leuB*, Δ*trpA*, Δ*cas6*) (this study) and H26Δ*cas*Cluster28 (Δ*pyrE2*, Δ*leuB*, Δ*trpA*, ΔpHV4:207.288–218.340) (42) were grown aerobically at 45 °C in Hv-YPC medium (42). *H. volcanii* strain H119Δ*cas6* containing plasmids with mutated *cas6* genes were grown in Hv-Ca medium supplemented with 0.25 mm tryptophan. *Escherichia coli* strains DH5α (Invitrogen) and GM121 (43) were grown aerobically at 37 °C in 2YT medium (44). *Halorubrum lacusprofundi* DL12 (45) was grown aerobically in Hv-YPC medium at 37 °C.

##### Construction of Plasmids and Transformation of H. volcanii

The 3×FLAG tag sequence was amplified by PCR using the oligonucleotides FLAGN and FLAG2 (46). The PCR fragment was digested with NdeI and HindIII and cloned into pTA927 (43), yielding pTA927-FLAG. The *cas6* gene was amplified by PCR from genomic DNA using the oligonucleotides Cas6FLAGNup and Cas6FLAGNdo (primer sequences are listed in supplemental Table 1). The resulting fragment was cloned into pBluescriptII (Stratagene) linearized with EcoRV, yielding pBlue-NFLAGcas6 (supplemental Table 2). This construct was used as a template to introduce the mutations, either by the QuikChange II site-directed mutagenesis kit (Agilent Technologies) or by inverse PCR using 5′-phosphorylated oligonucleotides followed by religation of the PCR fragment, using oligonucleotides carrying the desired mutation in both cases (supplemental Table 1). The resulting pBluescriptII constructs carrying the wild type *cas6* gene and mutated *cas6* genes were digested with HindIII and EcoRI, and the *cas6* gene variants were subcloned into the plasmid pTA927-FLAG, yielding the constructs pTA927-*cas6* and pTA927-*cas6*mutX, respectively (supplemental Table 2). The following constructs were generated to complement the strain H26Δ*cas*Cluster28 with different sets of *cas* genes expressed from *H. volcanii* vector pTA927: pTA927-Cas6875, pTA927-Cas685, pTA927-Cas687, and pTA927-Cas675. pTA927-Cas6875 was generated by PCR amplification of *cas6*, *cas8*, *cas7*, and *cas5* from genomic DNA of the wild type strain H119 with oligonucleotides Cas6FLAGCup and 3-Cas5-BamHI, followed by digestion of the PCR fragment with NdeI and BamHI and its subsequent cloning into pTA927. pTA927-Cas685 was generated by PCR amplification using oligonucleotides Cas6FLAGCup and 3-Cas5-BamHI and genomic DNA from an H119Δ*cas7* strain as a template, followed by digestion of the PCR fragment with NdeI and BamHI and its subsequent cloning into pTA927. pTA928-Cas687 was generated by PCR amplification using oligonucleotides Cas6FLAGCup and 3-Csh2-HindIII and genomic DNA of the wild type strain H119, followed by digestion of the PCR fragment with NdeI and HindIII and its subsequent cloning into pTA927. pTA927-Cas675 was generated by PCR amplification using oligonucleotides Cas6FLAGCup and 3-Cas5-BamHI and genomic DNA from the H119Δ*cas8* strain as a template, followed by digestion of the PCR fragment with NdeI and BamHI and its subsequent cloning into pTA927. All plasmids were passaged through *E. coli* GM121 cells to avoid methylation and then introduced into *H. volcanii* strains H119Δ*cas6* or H26Δ*cas*Cluster, respectively, using the polyethylene glycol method (47, 48). Transformants were selected on Hv-Ca plates without uracil.

##### Northern Blot Hybridization

Total RNA was isolated from exponentially growing *H. volcanii* cells as described (49) or isolated from the FLAG-Cas7 purified protein fraction as described below. After separation of 10 μg of RNA (total RNA) or 600 ng (FLAG-Cas7 fraction) on 8% denaturing gels, RNA molecules were transferred to nylon membranes (Hybond-N^+^, GE Healthcare) and incubated with oligonucleotides against the different spacers (primer P1.1, which is complementary to the sequence of the first spacer of locus P1; primer P1.2, which is complementary to the sequence of the second spacer of locus P1; primer P2.1, which is complementary to the sequence of the first spacer of locus P2; and primer C1, which is complementary to the sequence of the first spacer of locus C); as an RNA loading control, a hybridization with oligonucleotide 5 S, which binds to the 5 S rRNA was performed. All primers were radioactively labeled at the 5′-end with [γ-^32^P]ATP. To quantify the amount of mature crRNA, the membranes were exposed to imaging plates (BAS-MS, Fujifilm) and analyzed using the FLA-3000 scanner (software BASreader version 3.14). The intensity of the signals was measured with ImageJ. The signals of the P1.1 detection were put into relation to the 5 S rRNA signal, which was used as RNA loading control. To obtain the percentage of mature crRNA in the mutants, the signal of complementation of the *cas6* deletion strain with the wild type *cas6* gene was set to 100%, and the crRNA amounts of the *cas6* mutant strains were set in relation to these data. All Northern analyses were performed at least three times. In the analysis of the crRNA amounts with the Δ*cas*Cluster strain, the crRNA signals in the wild type strain were set to 100%, and the Δ*cas*Cluster strains transformed with the different sets of *cas* genes were set in relation to the wild type crRNA amounts.

##### RT-PCR and 5′-RACE

RNA was isolated as described above, and 1 μg was subjected to several reverse transcription reactions with primers Csh1#2, Csh2#2, Cas5#2, Cas3#5, Cas4#2, Cas1#5, and Cas2#2. The resulting cDNA was amplified with primers used for RT-PCR and primers Cas6#4, Csh1#3, Csh2#3, Cas5#3, Cas4#4, and Cas1#4. PCR fragments were cloned into pBluescriptII (digested with SmaI), and resulting clones were sequenced. For the 5′-RACE, 1 μg of RNA treated with 5′-phosphate-dependent exonuclease (Epicenter) to enrich primary transcripts was reverse transcribed with primer Cas6#3 using the MINT universal kit (Evrogen) according to the manufacturer's instructions. For the PCR, primer M1 from the MINT kit and primer Cas6#2 were used. PCR fragments were cloned into pBluescriptII (digested with SmaI), and resulting clones were sequenced.

##### Expression of cas6 in E. coli and H. volcanii

To express the *Haloferax cas6* in *E. coli*, the gene was amplified by PCR using the oligonucleotides 5-Cas6-NcoI and 3-Cas6-NotI. The fragment was digested with NcoI and NotI and cloned into the vectors pET28a and pET32a (Novagen), yielding the constructs pET28a-*cas6* (N-terminal His_6_ tag) and pET32a-*cas6* (N-terminal His_6_, S, and Trx tag). For the construct pET30a-*cas6*-FLAGN, the *cas6* gene together with the N-terminal 3×FLAG tag was removed from the construct pTA927-*cas6* by digestion with NdeI and EcoRI and cloned into the vector pET30a (digested with NdeI and EcoRI). To obtain a construct with a C-terminal tag, a PCR was performed using the oligonucleotides 5-Cas6-NcoI and 3-Cas6-HindIII, followed by digestion of the resulting fragment with NcoI and HindIII and cloning into the vector pET30a, yielding pET30a-*cas6*o.stop (C-terminal His_6_ tag). To improve the expression, the *cas6* gene was optimized to the *E. coli* codon usage by GeneArt (Invitrogen). The optimized gene was isolated from the original plasmid by digestion with NcoI and XhoI and cloned into different pET vectors, yielding the constructs pET28a-*cas6*h and pET30a-*cas6*h. *E. coli* strains BL21AI and BL21 codon plus (Novagen) were transformed with the respective constructs, and the protein was expressed according to the manufacturer's protocol. To improve the solubility of the recombinant protein, several different expression conditions (growth temperature and the amount of isopropyl 1-thio-β-d-galactopyranoside and/or arabinose for induction) were tested. To express the *cas6* gene in *Haloferax* cells, the strain H119Δ*cas6* was transformed with the construct pTA927-*cas6* (see above). To obtain a construct with His_6_ tag, the *cas6* gene was amplified by PCR, using the oligonucleotides 5-Cas6-PciI and 3-Cas6-NotI. The fragment was digested with PciI and NotI and cloned into the vector pTA963 (43), yielding the construct pTA963-*cas6*. The *Haloferax* strain H1424 (50) was transformed with this construct. To express the tagged Cas6 protein, the respective strains were grown in Hv-Ca medium containing 0.25 mm tryptophan. At exponential growth phase, tryptophan was added to a final concentration of 3 mm, and the culture was grown for an additional 2–16 h. To prepare soluble extracts, the cells were harvested and washed in enriched PBS buffer (2.5 m NaCl, 150 mm MgCl_2_, 1× PBS (137 mm NaCl, 2.7 mm KCl, 8 mm Na_2_HPO_4_, 2 mm K_2_HPO_4_, pH 7.4)). The cells were resuspended in lysis buffer (1 m NaCl, 100 mm Tris/HCl, pH 7.5, 1 mm EDTA, 10 mm MgCl_2_, 1 mm CaCl_2_, 8 units/μl DNase RQ1 (Promega), 13 μl/ml protease inhibitor mixture (Sigma)) and lysed by ultrasonification. Insoluble cell debris was removed by centrifugation at 48,000 × *g*. For affinity purification via the 3×FLAG tag, anti-FLAG M2 affinity gel (Sigma) was equilibrated with ice-cold washing buffer (50 mm Tris/HCl, pH 7.5, 1 m NaCl) and added to the extract. After incubation overnight at 4 °C, the affinity gel was washed extensively with washing buffer, and the protein was eluted by washing buffer to which 3×FLAG peptide (Sigma) was added to a final concentration of 150 ng/μl. His purification was performed according to the manufacturer's protocol with Protino nickel-nitrilotriacetic acid-agarose (Macherey-Nagel), using buffers with different concentrations of NaCl from 300 mm to 2 m.

##### Purification of a FLAG-tagged Cas7 Protein

To obtain the construct pTA927-Cas68[HisFLAG]75, which contains all four *cas* genes *cas5–8* with the *cas7* gene fused to cDNAs for a His and a FLAG tag, first the 3×FLAG sequence was amplified by PCR using the primers FLAGSnaBI and FLAG2. The product was digested with SnaBI and HindIII and cloned into the vector pCDF1b (Novagen) digested with PmlI and HindIII, resulting in the construct pCDF1b-FLAG. This construct was digested with XhoI, treated with *Pfu* polymerase to generate blunt ends, and then digested with HindIII. The genes *cas7* and *cas5* were amplified from genomic *Haloferax* DNA using primers 5-Csh2-HindIII and 3-Cas5-BamHI, and the resulting fragment was digested with HindIII and cloned into the pCDF1b-FLAG vector, yielding the construct pCDF1b-75HisFLAG. The genes for Cas7 and Cas5 together with the N-terminal His_6_ and 3×FLAG tag were amplified from this construct using the primers HisFLAGBamHI and 3-Cas5-XbaI and digested with BamHI and XbaI. The genes *cas6* and *cas8b* were amplified by PCR from genomic *Haloferax* DNA with the primers Cas6FLAGCup and 3-Cas8-NcoIBamHI, digested with NdeI and BamHI, and cloned into the vector pTA927, digested with the same enzymes. This construct was digested with BamHI and XbaI, and the insert Cas75HisFLAG was ligated into it, yielding the construct pTA927-Cas68[HisFLAG]75. The sequence was verified by sequencing, and subsequently *Haloferax* strain H26*cas*Cluster28 was transformed with the plasmid. Cells were grown to exponential phase in HvCa medium containing 0.25 mm tryptophan. Expression of the *cas* genes and FLAG purification were performed as described above for *cas6* gene expression in *Haloferax*. The resulting purified protein fraction was analyzed using SDS-PAGE and mass spectrometry.

##### Isolation of RNA from the FLAG-Cas7 Purified Fraction

The FLAG-Cas7 purified fraction was incubated with 20 μg of proteinase K for 30 min at 37 °C in 100 μl of buffer (100 mm Tris/HCl, pH 7.5, 12.5 mm EDTA, 150 mm NaCl, 0.2% SDS); subsequently, the solution was extracted with phenol/chloroform/isoamylalcohol. RNA was precipitated from the aqueous phase, and the resulting pellet was dissolved in water. The RNA fraction was analyzed using a Northern blot as described above.

##### Mass Spectrometry

For mass spectrometric analysis, proteins were in-gel-digested with trypsin as described previously (51). Extracted peptides were analyzed by liquid chromatography tandem mass spectrometry (LC-MS/MS) on an Orbitrap XL instrument (Thermo Fisher Scientific) under standard conditions. Peptide fragment spectra were searched against a target decoy database for *H. volcanii* (52), using MASCOT as a search engine. Peptides with a peptide score lower than 20 were not considered specific.

##### Quantification of Cascade Subunits Using Intensity-based Absolute Quantification (iBAQ)

0.25 μg of the proteins co-purified with FLAG-Cas7 as described above were dried in an Eppendorf tube and dissolved in 10 μl of aqueous solution of Universal Proteomics Standard 2 (UPS2; Sigma-Aldrich) in 1% Rapigest (Waters) to obtain a nominal protein concentration ratio of 0.25:1 μg of total (Cas proteins/UPS2). The sample was reduced with dithiothreitol (10 μl, 50 mm in 100 mm triethylammonium bicarbonate, 37 °C, 1 h), alkylated with iodacetamide (10 μl, 100 mm in 100 mm triethylammonium bicarbonate, 37 °C, 1 h), diluted with 70 μl of 100 mm triethylammonium bicarbonate, and digested with porcine trypsin (Promega; 1:20, 37 °C, 16 h). Following digestion, the Rapigest detergent was cleaved by acidifying the solution with trifluoroacetic acid (20 μl, 5%, 37 °C, 2 h) and pelleting the released fatty acids by centrifugation (13,000 rpm, 30 min, room temperature). The supernatant containing tryptic peptides was transferred to a fresh tube, taken to dryness in a SpeedVac, and dissolved in LC-MS loading buffer (30 μl, 5% acetonitrile, 0.1% formic acid). Samples were analyzed in triplicate (3 × 5 μl) on a nanoflow liquid chromatography system (1100 series, Agilent) coupled to an LTQ-Orbitrap Velos mass spectrometer in a vented column setup at an analytical flow rate of 300 nl/min achieved through passive splitting. Samples were desalted on an RP-C18 precolumn (20 mm, 0.15-mm inner diameter, ReproSil-Pur C18-AQ 5 μm; Dr. Maisch). Separation was achieved on an RP-C18 column (150 mm, 0.075-mm inner diameter, ReproSil-Pur C18-AQ 3 μm; Dr. Maisch) packed into a SilicaTip emitter (FS360-75-10-N, New Objective) using a 50-min linear gradient of 3–36% acetonitrile with 0.1% formic acid as modifier). MS data were acquired using a Top8 method with CID fragmentation, using a normalized collision energy of 45%.

Results were analyzed using MaxQuant software version 1.2.7.4 (53). The MS data were matched against an *H. volcanii*-filtered UniProt/trEMBL protein sequence database (version 2013-11) supplemented with the sequences of the 48 proteins contained in the UPS2 standard. iBAQ values from replicate values were averaged, and the S.D. value was calculated to judge the precision of analysis (supplemental Table 3). A total of 20 UPS2 standard proteins were observed in three of three replicate analyses and used for calibration. A calibration curve was obtained by linear regression from a double logarithmic plot (log(iBAQ) *versus* log(amount); supplemental Fig. 1). The calibration function was then used to calculate the respective Cas5-7 amounts in the co-purified fraction.

##### Generation of a cas6 Gene Deletion Strain

The deletion of the *cas6* gene was achieved by using the pop-in/pop-out method as described previously (43, 44, 46). The *cas6* gene was PCR-amplified with flanking regions (∼500 base pairs each) from the chromosomal DNA of *H. volcanii* strain H119 using primers Cas6KOup and Cas6KOdo. The resulting 1915-nt PCR fragment was subsequently cloned into the vector pTA131 (EcoRV), yielding pTA131-cas6geneupdo. To remove the *cas6* gene, an inverse PCR was performed on pTA131-cas6geneupdo with primers IPCas6up and IPCas6do2; the resulting linear PCR product was ligated, yielding pTA131-cas6updo. Plasmids were passaged through *E. coli* GM121 to prevent methylation, and *H. volcanii* strain H119 was subsequently transformed with this construct to allow integration (pop-in) of the plasmid into the genome. The subsequent selection for loss of the *pyrE2* marker by plating on 5-fluoroorotic acid revealed pop-out mutants. Chromosomal DNA was isolated from the wild type and potential *cas6* deletion mutants. Southern blot hybridization was performed as described (47), with the following modifications: 10 μg of SalI-digested DNA was separated on a 0.8% agarose gel and transferred to a nylon membrane (Hybond^TM^-N, GE Healthcare). The 500-base pair *cas6* downstream region was amplified using primers IPCas6KOdo2 and Cas6KOdo, labeled using the PCR DIG Probe Synthesis Kit (Roche Applied Science), and used as a hybridization probe. Hybridization and detection were performed according to the DIG manual (DIG Luminescent Detection Kit, Roche Applied Science).

##### Cloning of the Heterologous cas6 Genes

The *cas6* gene from *Methanosarcina mazei* was isolated from the original plasmid pRS714 (13) by PCR using primers Cas6Mmazfwd and Cas6Mmazrev. After digestions with NdeI and EcoRI, the gene was cloned into pTA927 (digested with NdeI and EcoRI), yielding pTA927-Mmazcas6. The *cas6* gene from *M. maripaludis* was isolated from the original plasmid pHR6 (16) by digestion with NdeI and HindIII and was cloned into the pTA927 vector, which was previously digested with NdeI and HindIII, thus yielding pTA927-Mmarcas6. The *cas6* genes Hlac3333 and Hlac3572 from *H. lacusprofundi* were ordered from Geneart (Invitrogen). Clones for Hlac3333 and Hlac3572 were digested with NcoI and BamHI and with NcoI and EcoRI, respectively, and resulting fragments were then cloned into vector pTA1228, yielding pTA1228-Hlac3333 and pTA1228-Hlac3572. pTA1228 is an improved version of the pTA963 overexpression plasmid (43). It features a unique NspI site in the polylinker (downstream of the His_6_ tag). This NspI site is compatible with SphI ends and allows for the cloning of genes where the second codon starts with C. pTA1228 was derived from pTA963 by the removal of two existing NspI sites. The NspI site (bp 1033) located between the pBluescript backbone and the pHV2 origin was inactivated by cutting with NsiI and filling in with Klenow. The NspI site (bp 7165) located in the *hdrB* selectable marker was inactivated by replacement of a 762-bp BspEI-NcoI fragment (comprising the 5′ part of *hdrB* and the 3′ part of *pyrE2*) with a nearly identical 762-bp BspEI-NcoI PCR fragment that features a synonymous Gly → Ala mutation (GAC → GAT) at the NspI site. The internal hdrB-dNsp primers with the Gly → A mutation were hdrB-dNsp F and hdrB-dNsp R. The sequence and map of pTA1228 are available upon request.

##### Modeling of cas6 Three-dimensional Structure

To obtain a prediction of the tertiary structure of the *Haloferax* Cas6 protein, the amino acid sequence was submitted to the Phyre server (protein homology/analogy recognition engine) (54). According to the Phyre database, the *Pryrococcus* Cas6 protein showed the closest related structure. The three-dimensional structure was modeled with the help of the graphical software PyMOL.

##### Interference Tests

*H. volcanii* strain H119Δ*cas6* was transformed with the plasmids containing the *cas6* wild type gene (pTA927-*cas6*) or the *cas6* mutants (pTA927-*cas6*mutX). In a second transformation, these strains were transformed with the invader plasmid pTA352-PAM3 or pTA352-PAM9 (supplemental Table 2) (42). Transformants were selected on Hv-Min plates without leucine and uracil. As a positive control, the respective strains were transformed with the vector pTA352. To confirm a successful interference reaction, *H. volcanii* cells were transformed at least three times with one of the plasmid invader constructs. As has been observed in similar studies (42, 55), it is difficult to accurately determine transformation rates; therefore, we defined only those sequences that led to at least a 100-fold reduction in transformation rates (reduced by a factor of 0.01) in this plasmid assay as a successful interference reaction.

## RESULTS

### 

#### 

##### The Family of Cas6 Proteins and the Haloferax Cas6 Protein

**FIGURE 2.**
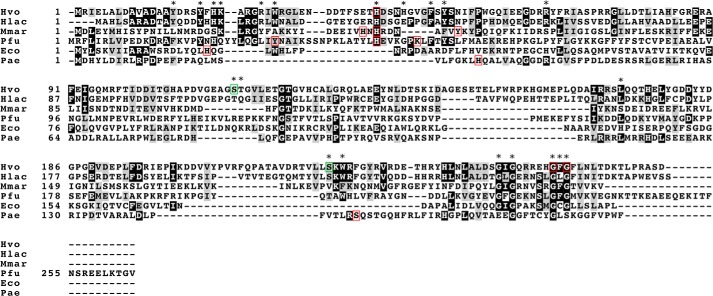
**Alignment of Cas6 proteins.** Cas6 proteins from *Haloferax*, *H. lacusprofundi*, *M. maripaludis*, *P. furiosus*, *E. coli*, and *P. aeruginosa* are aligned to identify conserved amino acids. Amino acids from *M. maripaludis*, *P. furiosus*, *E. coli*, and *P. aeruginosa* that were shown to be essential for catalysis are indicated by *red boxes*. The amino acids mutated in this study are indicated by *asterisks*. Amino acids shown to be important for the stable crRNA population in this study are *boxed* in *red* (mutations result in reduced crRNA amounts) or *green* (mutations result in higher crRNA amounts).

**FIGURE 3.**
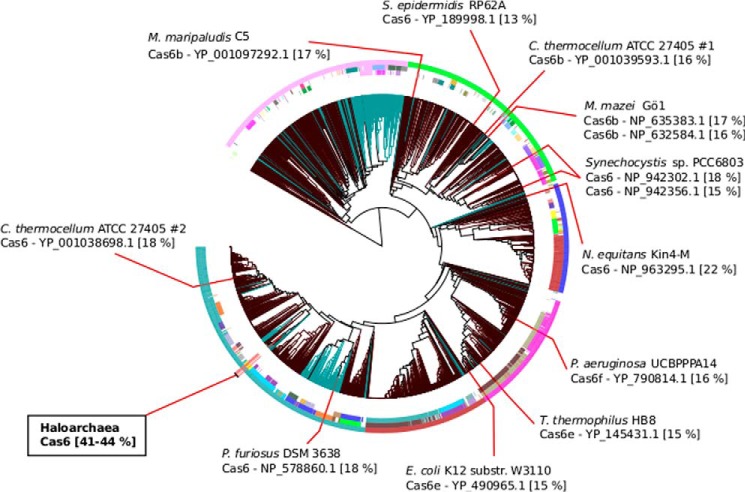
**The haloarchaeal CRISPR-Cas systems are different from other systems.** The haloarchaeal CRISPR-Cas systems are distinct from published systems where the Cas6 protein has been functionally characterized. The circular hierarchical tree represents the sequence and structure similarity of repeats from all publicly available genomes, taken from the CRISPRmap Web server (39). The locations of repeats associated with previously characterized Cas6 are highlighted with *red lines*: *Clostridium thermocellum* (16), *P. furiosus* (10, 31, 32), *E. coli* (20), *T. thermophilus* (25, 27, 29), *P. aeruginosa* (21, 26), *Nanoarchaeum equitans* (65), *Synechocystis* (66), *M. mazei* (13), *S. epidermidis* (30), and *M. maripaludis* (16). The pairwise alignment percentage identities in comparison with the Cas6 protein in *H. volcanii* are given in *square brackets*. For the CRISPRmap tree, *brown branches* represent CRISPRs from bacteria, the *blue-green branches* represent CRISPRs from archaea, the *inner annotation circle* represents different conserved structure motifs, the *middle circle* represents conserved sequence families, and the *outer circle* represents the six superclasses.

The *Haloferax* Cas6 protein shows very little sequence similarity to other Cas6 proteins from type I systems and to most systems in general (Fig. 2). As visible in the alignment, only three positions are conserved in all Cas6 proteins analyzed (Gly-122, Gly-258, and Gly-258). Only one amino acid, shown to be important for catalytic activity in other organisms, is conserved in the *Haloferax* Cas6 protein (His-41). To analyze the family of Cas6 proteins and to determine Cas6 protein conservation, we first performed a BLAST search with the *H. volcanii* Cas6 (YP_003533663.1) and subsequently quantified similarity via pairwise global sequence alignments (using the NCBI implementation of the Needleman-Wunsch algorithm (56)). Close homologs of the *Haloferax* Cas6 protein were only found in closely related Haloarchaea with global percentage identities between 41 and 44% and BLAST *E*-values of <1 × 10^−60^, including *Haloferax mediterranei* (ATCC 33500), *Haloarcula marismortui* (ATCC 43049), *Halorhabdus utahensis* (DSM 12940), *H. lacusprofundi* (ATCC 49239), *Natrinema* (sp. J7-2), and *Halomicrobium mukohataei* (DSM 12286). Apart from the Haloarchaea, the similarity to other Cas6 proteins is very low, with pairwise similarities of only 13–22% to other Cas6-like proteins (Fig. 3). Even the Cas6 protein from *P. furiosus*, which of all of the Cas6 proteins investigated is the closest to *H. volcanii*, has an equally low similarity of 18%. The Cas6 proteins from other systems show comparably low levels of sequence similarities with each other.

Conservation of the *H. volcanii* repeats is analogous to the conservation of the Cas6 protein; the *Haloferax* repeat sequences are very different from other published systems, except in Haloarchaea. Repeats from haloarchaeal CRISPR-Cas systems are, in fact, well conserved and can form a minimal hairpin structure with three base pairs (23). When using the Web server CRISPRmap (39), which compares all available repeats according to sequence and structure similarity, the haloarchaeal CRISPR repeats cluster together into the sequence family F19. The uniqueness of the haloarchaeal family F19 is highlighted by the fact that it is located on an isolated branch of the hierarchical CRISPRmap tree of all repeat sequences (Fig. 3). Thus, in the large family of Cas6-like proteins, not only is the sequence similarity low, but the individual proteins are highly specific to their associated repeat sequences and have different functional mechanisms. Obviously, the full spectrum of this family has not yet been explored. Therefore, we analyzed the *Haloferax* Cas6 protein in detail to provide insights into this extremely diverse family.

##### cas Genes Show a Low Level of Expression

Earlier studies showed that the CRISPR RNAs are constitutively expressed in *Haloferax* (42). To analyze whether the *cas* genes are likewise constitutively expressed, we used Northern blot analyses to determine the amount and the length of the *cas* mRNAs. No signals were detected for the *cas* mRNAs on Northern blots with RNA isolated from cells grown under different conditions (standard conditions, low and high temperatures, low and high salt concentrations) (data not shown). Therefore, we used RT-PCR to determine if the genes are transcribed at all, revealing that the mRNA for all *cas* genes is present (data not shown). Employing 5′-RACE, we determined the 5′-end of the *cas6* mRNA, which is located 39 nucleotides upstream of the Cas6 protein start codon. Expression of the *cas* genes was confirmed in a parallel study in which the *Haloferax* proteome was investigated.^3^ Here, it was shown that all eight Cas proteins were present in the proteome in both the exponential and stationary phases. Taken together, we could show that the *cas* mRNA is present, albeit in low amounts.

##### Cas6 Is Necessary for crRNA Production

**FIGURE 4.**
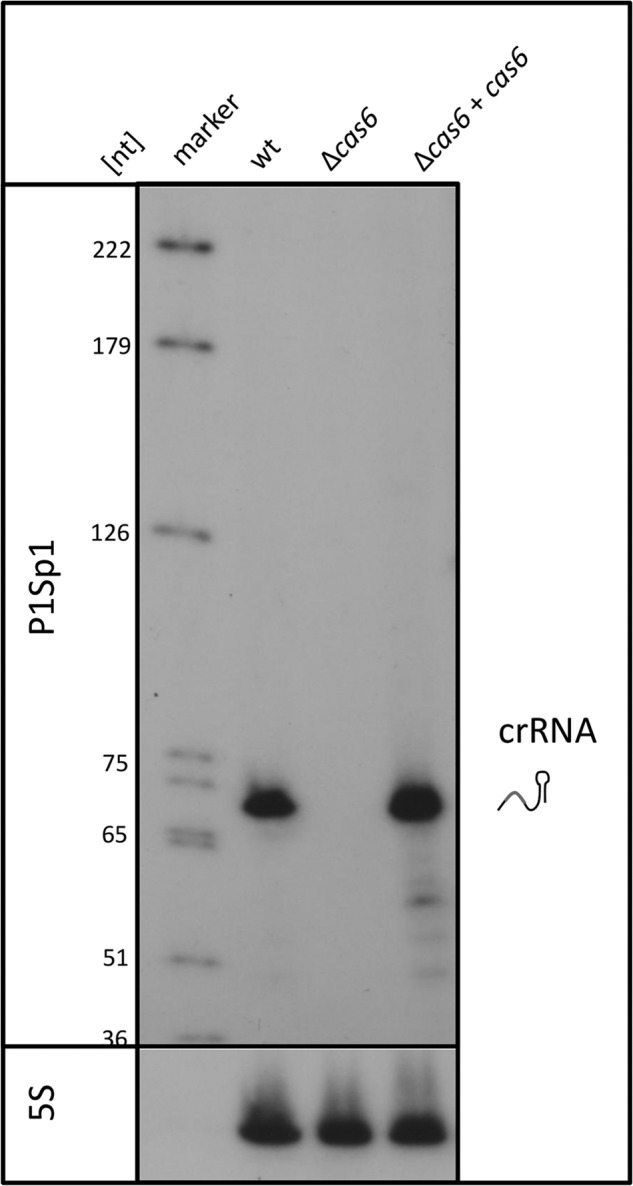
**The *cas6* deletion strain does not generate crRNAs.** To determine the biological function of the Cas6 protein, we deleted the reading frame of this gene in *H. volcanii* and isolated RNA from the deletion (Δ*cas6*) and wild type strains, which was subsequently separated on 8% PAGE and transferred to a membrane. Hybridization with a probe against spacer 1 from locus P1 showed that no crRNAs were generated in the Δ*cas6* strain. Complementation of this strain with the *cas6* gene on a plasmid resulted in the rescue of crRNA production.

To investigate the biochemical characteristics of the Cas6 protein, we expressed recombinant Cas6 protein in *E. coli* and *H. volcanii* using different approaches (for details, see “Experimental Procedures”). The *cas6* gene was cloned into different expression vectors, and *Haloferax* and different *E. coli* strains were transformed with these constructs. All attempts to produce an active recombinant Cas6 protein failed; therefore, we next used an *in vivo* approach to investigate the Cas6 protein. To determine the *in vivo* function of Cas6 in *Haloferax*, we generated a *cas6* deletion strain. Because the *cas6* gene overlaps with one nucleotide of the downstream *cas8b* gene (Fig. 1), we deleted the complete reading frame with the exception of the last nucleotide that is shared with the downstream *cas8b* gene. The *cas6* deletion strain showed no visible phenotype in comparisons with the respective wild type strain when grown under different conditions, such as different temperatures and salt concentrations (data not shown). To investigate whether *Haloferax* is able to produce mature crRNAs without the Cas6 protein, we isolated RNA from wild type and *cas6* deletion strains and analyzed the amount of crRNAs by Northern blot. Hybridization with probes against spacer 1 from locus P1 (Fig. 4) and spacers from the other CRISPR loci (P1.2, P2.1, and C1) (data not shown) revealed that the wild type strain contains mature crRNAs, but the deletion strain does not. To confirm that the failure to produce or stabilize mature crRNAs is due to the missing Cas6 protein and not to some effect on the downstream-encoded *cas* genes, we complemented the *cas6* deletion strain with the FLAG-Cas6 overexpression construct (described under “Experimental Procedures”) (Fig. 4). Complementation restores the wild type amount of crRNAs, verifying that Cas6 is required for efficient crRNA production. To investigate whether *cas6* genes from other organisms would be able to complement the deletion mutant, we transformed the deletion mutant with the *cas6* genes from another halophilic archaeon, *H. lacusprofundi.* Northern analysis showed that these genes are not able to complement the deletion mutant strain (data not shown). Furthermore, we complemented the *cas6* gene deletion mutant with type I-B *cas6* genes from two non-halophilic archaea, *M. maripaludis* (16) and *M. mazei* (13). Neither of the two heterologous *cas6* genes was able to complement the *Haloferax cas6* gene deletion mutant (data not shown). The repeat sequences of the CRISPR RNAs from the four different organisms show almost no sequence similarities, which might be the reason that the heterologous Cas6 proteins cannot process the *Haloferax* repeat. Taken together, we could show that without the *cas6* gene, crRNAs cannot be generated or maintained *in vivo* in *Haloferax*.

##### Single Amino Acid Mutations in Cas6 Reveal both a Loss and a Gain in crRNA Levels

**FIGURE 5.**
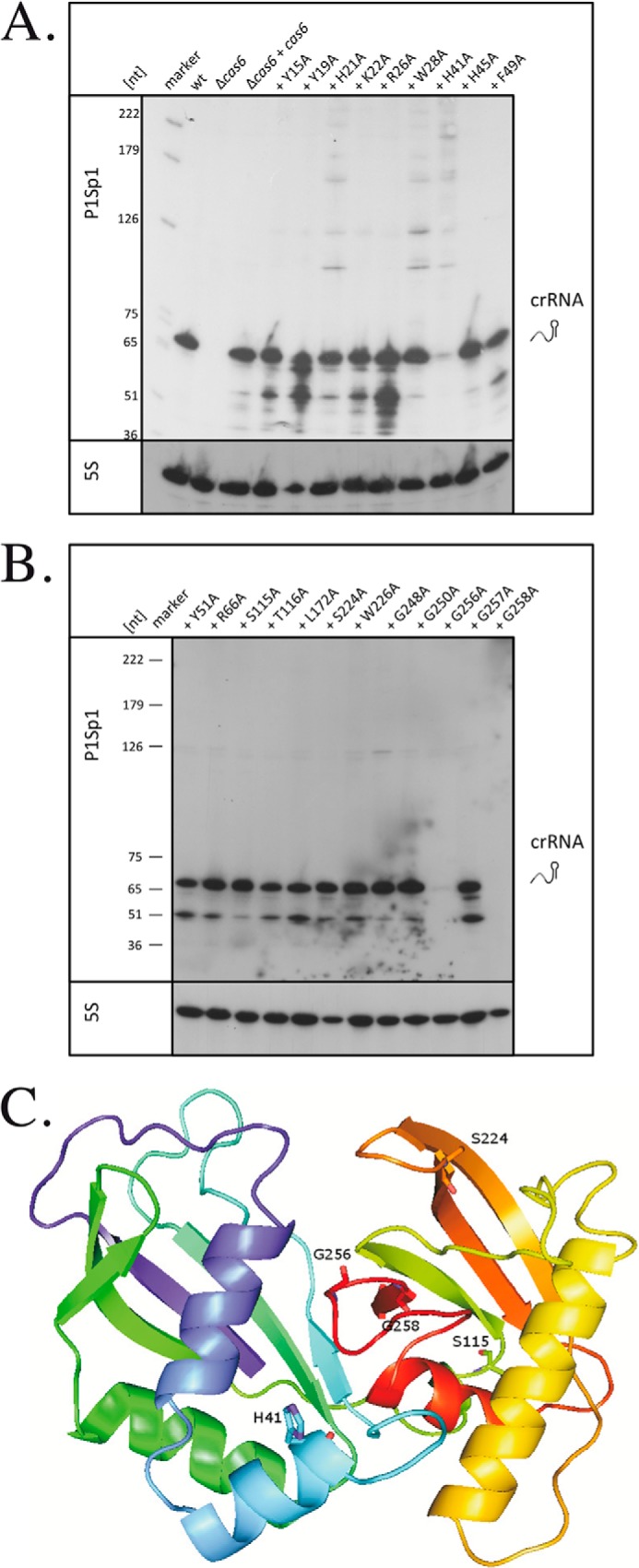
**Effect of *cas6* gene mutations on crRNA amounts.** Mutations were introduced into the *cas6* gene (Fig. 2), and the *cas6* deletion strain was transformed with the mutant genes. *A* and *B*, RNA from all strains was isolated, separated on 8% PAGE, and subsequently transferred to Northern membranes that were then hybridized with a probe against spacer 1 from locus P1. Determination of the amount of crRNA in relation to the amount of RNA loaded (measured by the 5 S rRNA hybridization) showed that only transformation with three variants (His-41, Gly-256, and Gly-258) resulted in reduced amounts of crRNAs. Higher amounts of crRNA were determined in mutants Ser-115 and Ser-224. On the *left*, a DNA size marker in nucleotides is shown. The hybridization with the spacer 1 from CRISPR locus P1 is shown at the *top*, and hybridization with a probe against 5 S rRNA is shown at the *bottom. marker*, the DNA size marker; *wt*, RNA from wild type cells; Δ*cas6*, RNA from the *cas6* gene deletion strain; Δ*cas6* + *cas6*, RNA from the *cas6* gene deletion strain complemented with the wild type *cas6* gene from a plasmid; +*Y15A*, +*Y19A*, +*H21A*, +*K22A*, +*R26A*, +*W28A*, +*H41A*, +*H45A*, +*F49A*, +*Y51A*, +*R66A*, +*S115A*, +*T116A*, +*L172A*, +*S224A*, +*W226A*, +*G248A*, +*G250A*, +*G256A*, +*F257A*, and +*G258A*, RNA from the *cas6* deletion strain complemented with the mutant *cas6* gene from a plasmid. *C*, model of the *H. volcanii* Cas6 protein. Similarity searches in the Phyre database (54) show the closest structures related to the *H. volcanii* Cas6 protein to be the *P. furiosus* Cas6 structure (32). The *H. volcanii* protein was modeled according to the published *P. furiosus* structure, and the amino acid mutations that changed the amounts of crRNA are shown. His-41 is located where the catalytic site in the *Pfu* Cas6 protein was proposed; according to this model, the two glycines and serines might be located on the surface where the crRNA could be located.

To identify amino acids necessary for generating or stabilizing crRNAs, we introduced 21 point mutations in the *cas6* gene to change the original amino acid to alanine (Fig. 2). We have chosen amino acids for mutation that, according to the alignment in Fig. 2, either directly align with amino acids shown in other Cas6 proteins to be important for function or are in the vicinity of such amino acids. In addition, amino acids that were shown to be conserved in at least three Cas6 proteins were chosen. The *Haloferax* H119Δ*cas6* strain was transformed with the 21 *cas6* mutant constructs, and RNA was subsequently isolated for Northern analyses. Hybridization with a probe against spacer 1 from the CRISPR locus P1 showed that most of the mutants were still able to generate the crRNA (Fig. 5 and Table 1). Only three mutations resulted in reduced (less than 50% of wild type amount) crRNA amounts. Changing the amino acid His-41 to Ala reduced the amount of crRNA to 37% of the wild type amount, whereas changing Gly-256 or Gly-258 reduced it to 12 and 0% of the wild type amount, respectively. Interestingly, we also observed a gain of function in two mutants (mutants Ser-115 and Ser-224) that resulted in higher crRNA amounts of 129 and 164%, respectively. To investigate whether the severely reduced crRNA levels in mutants Gly-256 and Gly-258 are due to parts of the protein being in the insoluble fraction due to improper folding, we isolated whole cell extract from the transformed cells and analyzed the insoluble and soluble protein fraction for the presence of the Cas6 protein mutants. Both Cas6 protein mutants were detectable in the soluble fraction, and the amount of protein did not change significantly (data not shown). Therefore, the reduced activity is not due to production of an insoluble protein.

**TABLE 1:** **Effect of amino acid changes on the amount of crRNA** The amounts of crRNA detectable in the wild type *H. volcanii* strain, the *cas6* gene deletion strain, the *cas6* gene deletion strain complemented with the wild type *cas6* gene, and the *cas6* mutants were determined and assessed relative to the amounts of 5 S rRNA detected (see “Experimental Procedures”). The amount of crRNA detected in the *cas6* deletion strain complemented with the wild type *cas6* gene on a plasmid was set to 100%.

Mutant	Processing activity	S.D.	Compared with wild type Cas6
	%		
WT	61	3	
Δ*6*	3	4	
Δ*6* + *6*	100		
Y15A	98	6	
Y19A	110	16	
H21A	69	5	
K22A	94	7	
R26A	100	7	
W28A	105	3	
**H41A**	**37**	**2**	**↓**
H45A	81	7	
F49A	84	1	
Y51A	71	2	
R66A	104	2	
**S115A**	**129**	**6**	**↑**
T116A	89	2	
L172A	68	3	
**S224A**	**164**	**6**	**↑**
W226A	108	3	
G248A	89	5	
G250A	90	5	
**G256A**	**12**	**2**	**↓**
F257A	111	3	
**G258A**	**0**	**0**	**↓**

##### Cas6 Mutations Can Abolish the Interference Reaction

Type I Cas6 proteins have been shown to be involved in the generation of crRNAs. They have also been shown to stay attached to the crRNA after processing. To determine whether an intact Cas6 is important for the interference reaction and which amino acids are important for this activity, we subjected all *cas6* mutants to an interference test, which we previously established in *Haloferax* by using a plasmid-based invader (42). The invader plasmid contains a protospacer that is recognized by one of the *Haloferax* crRNAs and PAM motifs that are recognized by the *Haloferax* CRISPR-Cas system and trigger the defense reaction. Plasmid invaders were introduced into a *Haloferax* strain that cannot grow without uracil because the strain lacks the *pyrE2* gene. Transformants were selected by uracil prototrophy conferred by the *pyrE2* gene on the plasmid invader. Plasmid elimination via the CRISPR-Cas defense of the host cell were detected by a drastically reduced transformation rate. Cells with an active interference system degrade the invader plasmid efficiently, reducing the transformation rate by at least a factor of 0.01. Transformation of the *cas6* deletion strain with the invader plasmid resulted in normal transformation rates, showing that deletion of the *cas6* gene abolishes the CRISPR interference reaction of the cells (Table 2). If the *cas6* deletion strain was complemented with the *cas6* wild type gene and then transformed with the invader plasmid, only a few transformants were obtained (the transformation rate was reduced by a factor of 0.01) (Table 2), showing that the interference was working again. Next, the *cas6* gene deletion strain was transformed, first with the mutated *cas6* genes and then with the invader plasmid, to investigate which Cas6 protein mutations would interfere with the defense reaction. Three mutants showed reduced or no interference reactions: His-41, Gly-256, and Gly-258 (Table 2). Transformation of the *cas6* deletion strain with the His-41 mutant and the invader plasmid resulted in reduction of the transformation rate by only a factor of 0.2; the plasmids were not degraded very efficiently. Mutants Gly-256 and Gly-258 showed a normal transformation rate; thus, the interference reaction was not active and was not able to degrade the plasmid. The increased crRNA amounts in the Ser mutant strains did not change the behavior in the interference reaction. The interference reaction was working efficiently, reducing the transformation rate by a factor of at least 0.001 (Ser-224) and 0.002 (Ser-115) (Table 2). Taken together, we could show that *cas6* mutations, which reduce the crRNA amounts, likewise reduce the effectiveness of the interference reaction.

**TABLE 2:** **Cas6 mutations that result in reduced transformation rates** The *cas6* deletion strain was first transformed with the *cas6* wild type gene or the *cas6* mutants. Next, this strain was transformed with the invader plasmid (pTA352-PAM3 and pTA352-PAM9) to trigger the defense reaction. The invader plasmid contains the protospacer sequence that is detected by a crRNA from *Haloferax* and the PAM sequence (PAM3:TTC and PAM9:ACT) (42). The factor by which the transformation rate was changed was determined.

First transformation of Δ*cas6* with *cas6* gene and *cas6* gene mutants	Second transformation with invader plasmid	Reduction of transformation by factor
	pTA409-PAM3 and PAM9	No reduction (no interference)
pTA927-*cas6*	pTA352-PAM3 and PAM9	0.003 (interference restored)
pTA927-H41A	pTA352-PAM3 and PAM9	0.2 (reduced interference)
pTA927-S115A	pTA352-PAM3 and PAM9	0.002 (interference restored)
pTA927-S224A	pTA352-PAM3 and PAM9	0.001 (interference restored)
pTA927-G256A	pTA352-PAM3 and PAM9	No reduction (no interference)
pTA927-G258A	pTA352-PAM3 and PAM9	No reduction (no interference)

##### A Strain with Only cas6 Is Not Active in crRNA Production

**FIGURE 6.**
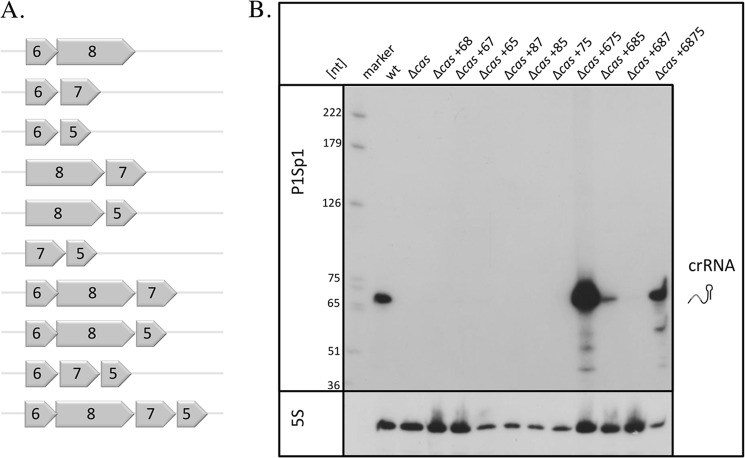
**Cas6 alone is not sufficient for crRNA processing and maintenance.** *A*, constructs used for complementation. To determine which *cas* genes are required for crRNA maturation, different *cas* gene constructs were generated for transformation of the H26Δ*cas*Cluster28 strain, which has the complete *cas* gene cluster deleted. *B*, effect of different Cas proteins on crRNA amounts. RNA isolated from the H26Δ*cas*Cluster28 strains, transformed with the different *cas* gene combinations, was transferred to membranes that were subsequently hybridized with a probe against spacer 1 from CRISPR locus P1. On the *left*, a DNA size marker (in nucleotides) is shown. The hybridization with spacer 1 from CRISPR locus P1 is shown at the *top*, and hybridization with a probe against 5 S rRNA is shown at the *bottom. marker*, DNA size marker; *wt*, RNA from wild type cells; Δ*cas*, RNA from the *cas* gene deletion strain; Δ*cas* + *68*, RNA from the *cas* gene deletion strain complemented with the *cas6* and *cas8b* genes from a plasmid; +*67*, +*65*, +*87*, +*85*, +*75*, +*675*, +*685*, +*687*, and +*6875*, RNA from the *cas* gene deletion strain complemented with the different *cas* genes from a plasmid as indicated.

Next, we investigated whether Cas6 alone is sufficient for crRNA maturation. We used a *Haloferax* strain in which all *cas* genes were deleted (H26Δ*cas*Cluster28 (42)) for transformation with the wild type *cas6* gene to investigate whether the Cas6 protein alone is sufficient for pre-crRNA processing and stabilization. Northern analysis of this strain showed no crRNA maturation, suggesting that the *cas6* gene is necessary for crRNA production (because deletion of the *cas6* gene results in loss of crRNA processing) but not sufficient for crRNA maintenance. Therefore, one or more of the other Cas proteins are also required. The Cas proteins that are potentially also involved in crRNA maintenance are Cas5, Cas7, and Cas8b, which are encoded together with Cas6 at the 5′-end of the *cas* gene locus (Fig. 1). To analyze whether the presence of all four of these genes rescues crRNA generation, we transformed strain H26Δ*cas*Cluster28 with the complete 5′ part of the *cas* gene cluster (encoding the Cas5–8b proteins) (Fig. 6*A*). RNA from this strain was analyzed with Northern blots for crRNA generation, revealing that crRNAs were generated and stably maintained (Table 3 and Fig. 6*B*). To determine if one of the four Cas proteins can be omitted, we next transformed the H26Δ*cas*Cluster28 strain with the genes for 1) Cas5, Cas6, and Cas7; 2) Cas5, Cas6, and Cas8b; and 3) Cas6, Cas7, and Cas8b. The combination of Cas6, Cas7, and Cas8b was not able to stably maintain crRNAs, whereas the combination of Cas5, Cas6, and Cas8b resulted in some amounts of crRNA (Table 3 and Fig. 6*B*). Only the combination of Cas5, Cas6, and Cas7 yielded normal crRNA amounts. We also tested all dimeric combinations of the Cas proteins 5–8b, but none of these were active in crRNA protection (Fig. 6*B*) (Table 3). Taken together, these results indicate that the Cas6 protein is necessary for generating crRNAs but not sufficient for its maintenance. *In vivo*, at least Cas5 and Cas7 are additionally required for crRNA generation or stabilization.

**TABLE 3:** **Cas proteins important for crRNA production and stability** The strain for which the complete *cas* gene cluster was deleted (H26ΔcasCluster28) was transformed with the different combinations of *cas* genes. The amount of crRNA was determined and assessed relative to the amount of 5 S rRNA detected (see “Experimental Procedures”). The amount of crRNA in the wild type strain was set to 100%.

Strain	crRNA amount
	%
WT	100
Δ*cas*	2.3
Δ*cas6* + *68*	2.4
Δ*cas6* + *67*	2.6
Δ*cas6* + *65*	3.2
Δ*cas6* + *87*	0
Δ*cas6* + *85*	0.4
Δ*cas6* + *75*	3.8
Δ*cas6* + *675*	96.8
Δ*cas6* + *685*	32.2
Δ*cas6* + *687*	8.0
Δ*cas6* + *6875*	97.9

##### A Cascade-like Complex in Haloferax

**FIGURE 7.**
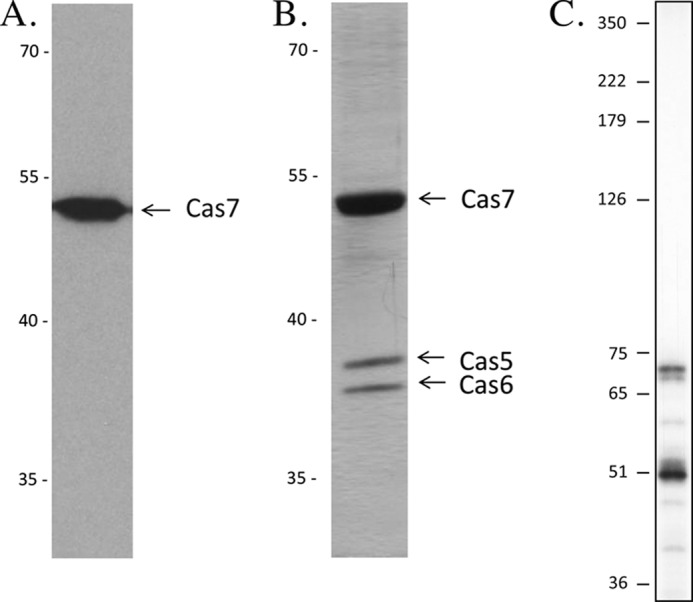
*A* and *B*, Cas5 and Cas6 co-purify with a FLAG-tagged Cas7 protein. A FLAG-tagged Cas7 protein was generated in *Haloferax* cells and purified together with all potential interaction partners using the FLAG tag. The purified fraction was investigated using a Western blot (*A*) and SDS-PAGE (*B*). Three proteins are visible on the Coomassie-stained SDS gel. The largest protein is, according to the Western blot (which was probed with the FLAG antibody), the FLAG-Cas7 fusion protein. According to mass spectrometry analyses, the two smaller proteins are Cas5 and Cas6, respectively. *A*, Western blot of the FLAG-purified fraction, probed with an anti-FLAG antibody from a plasmid; *B*, silver-stained SDS-PAGE of the FLAG-purified fraction. The protein size marker is given on the *left* in kDa, and the nature of the respective proteins is indicated on the *right. C*, the Cascade I-B complex contains crRNA. RNA was isolated from the FLAG-Cas7 purified protein fraction, separated by 8% PAGE, and transferred to a membrane. Hybridization with a probe against spacer 1 from CRISPR locus P1 detected two RNAs of ∼68 nucleotides and 51 nucleotides in length, which correspond to the *Haloferax* crRNAs (42). Shown on the *left* is a DNA size marker in nucleotides.

To confirm an association of the Cas5, Cas6, and Cas7 proteins, we expressed a FLAG-Cas7 fusion protein and purified this protein together with all potential interaction partners (Fig. 7). The SDS gel of the purified protein fraction shows co-purification of two additional proteins. Mass spectrometry analyses of the proteins showed that these proteins are Cas5 and Cas6. To further investigate the stoichiometry of Cas5, Cas6, and Cas7 in the complex by mass spectrometry, we used the iBAQ quantification approach (53, 57). The isolated complex was spiked into a mixture of quantified standard proteins (UPS2), which spanned a concentration range of 5 orders of magnitude, and the complete protein mixture was then digested in solution. The iBAQ values obtained for the Cas proteins were calibrated using a linear function derived from the iBAQ values for the standard proteins. The derived protein concentrations indicate a Cas5/Cas6/Cas7 stoichiometry of 1.7:1:8.5. This low/low/high type stoichiometry is generally in agreement with stoichiometries previously observed for Cascade-type protein complexes in *E. coli* (58) and *P. aeruginosa* (24). It should be noted that bottom-up mass spectrometric methods like iBAQ show limited accuracy for determining high stoichiometries in protein complexes because the ratio accuracy is largely limited by the accuracy of determining the higher protein copy numbers in complexes.

To investigate whether crRNAs are likewise part of the complex, we isolated RNA from the FLAG-Cas7 purified protein fraction and determined the nature of the RNA using a Northern blot (Fig. 7*C*). Hybridization with a probe against spacer 1 from CRISPR locus P1 showed that the RNA is indeed crRNA. In addition to the crRNA of about 68 nucleotides in length, a shorter crRNA of about 51 nucleotides is detected, suggesting a secondary processing event. These shorter crRNAs are also visible in other Northern analyses (Fig. 6) (42) but not as prominent as in the FLAG-Cas7 purified fraction. In summary, we could show that Cas5, Cas6, and Cas7, as well as crRNA, co-purify and that this Cascade-like complex has a similar composition as the *E. coli* Cascade complex.

## DISCUSSION

The Cas6 family of proteins is very diverse; only some structural elements, such as the ferredoxin fold and the glycine-rich loop, are conserved. The binding of the pre-crRNA and the catalytic site, however, differ from protein to protein. To learn more about this protein family, more data are required regarding several additional Cas6 proteins. We report here the first *in vivo* systematic analysis of Cas6 protein mutants that investigates the effect of the mutations on the crRNA amount and the interference reaction.

Only 3 of 21 tested mutations had a severe effect on the detectable crRNA amount: H41A, G256A, and G258A. Based on the alignment with other Cas6 proteins, His-41 is conserved in the archaeal Cas6 proteins from *M. maripaludis* (16) and *P. furiosus* (10), where it was shown to be important for catalysis in *in vitro* analyses. Whereas all of the Cas6 proteins investigated had a histidine essential for catalytic activity, one of the Cas6 proteins from *S. solfataricus* (Sso1437) has been shown to lack such a catalytic histidine (15, 17). Instead, several amino acids have been shown to be involved in catalysis: Lys-25, Lys-28, Lys-51, and Arg-231. Because *Haloferax* His-41 aligns with the catalytic histidines from the Cas6 proteins from *M. maripaludis* (16) and *P. furiosus* (10), it is likely that His-41 is also important for catalysis in *Haloferax.* According to the potential structure (Fig. 5*C*), where the *H. volcanii* Cas6 protein is modeled according to the *P. furiosus* Cas6 structure, His-41 is located at the proposed catalytic site.

The *in vitro* analyses with *M. maripaludis* and *P. furiosus* proteins illustrated the differences between these proteins because the additional amino acids identified to be important for *in vitro* catalysis were different: His-38 and Tyr-47 in *M. maripaludis* (16) and Tyr-31 and Lys-52 in *P. furiosus* (10). The additional histidines in *Haloferax* Cas6 (His-21 and His-45) were also mutated in this analysis, but no effect was detected. Likewise additional tyrosines and a lysine were mutated (Tyr-15, Tyr-19, Tyr-51, and Lys-22), but, again, the mutations did not change the amount of crRNA or interference. Therefore, *in vivo* either only His-41 is important for catalysis, or additional amino acids that were not mutated in this study are required.

Mutation of all four glycines in the glycine-rich loop of the *P. furiosus* Cas6 revealed that this mutant had no RNA cleavage activity anymore but maintained a weak RNA binding activity (32). The authors hypothesized that the path of the RNA on the Cas6 protein overlaps with the G-rich loop, explaining the effect of mutations in the glycine-rich loop. In *Haloferax*, two glycines from the glycine-rich loop are also important for generation of crRNAs or crRNA stability *in vivo*; the other two glycines are not important, and, likewise, neither is Phe-257, which is located in the glycine-rich loop between the two important glycines (Gly-256 and Gly-258). This finding might suggest that only the two important glycines (of all of the amino acids from the glycine-rich loop) interact with parts of the crRNA.

Two mutations, Ser-115 and Ser-224, resulted in a higher amount of crRNA. According to the structural model, the two serines might be located on the surfaces where the path of the crRNA is predicted to be in the *P. furiosus* Cas6 (Fig. 5*C*). This model would suggest an interaction of the two serines with the crRNA. A mutation in an amino acid important for crRNA binding might weaken product binding and thereby enhance product turnover, as observed in the *T. thermophilus* Cas6e protein (14, 25).

The experiments to reveal the impact of the mutations on the interference reaction showed that the amino acids identified as important for cleavage and crRNA stability were also important for interference. This result was expected because crRNAs are essential for the interference reaction. The H41A mutation resulted in reduced amounts of crRNA (37%) (Table 1), and the interference reaction was only reduced by a factor of 0.2. This might suggest that the amount of crRNA is important for an efficient interference reaction.

Amino acids that are not required for catalysis but might be important for the interaction of the Cas6 protein with the Cascade complex were not identified. A final conclusion on the nature of the catalytic site and binding residues of the Cas6 protein will only be reached by structure analysis of the *H. volcanii* Cas6 protein.

### 

#### 

##### The Cascade I-B Complex Requires Cas5, Cas6, and Cas7 for Maintaining a Stable crRNA Population

A *Haloferax* strain in which all *cas* genes, with the exception of *cas6*, have been removed is not able to stably maintain crRNAs. crRNAs can only be stably maintained if Cas5, Cas6, and Cas7 are present. This finding suggests the presence of a Cascade-like complex for crRNA processing and stabilization in the *H. volcanii* type I-B system, similar to the type I-A (Cascade I-A) and I-E (Cascade I-E) systems. The Cascade I-B complex clearly requires the Cas proteins 5–7 for maintaining a stable crRNA population. This hypothesis is confirmed by the co-purification of Cas5, Cas6, and crRNA molecules with a FLAG-tagged Cas7.

Interestingly, the combination of Cas proteins Cas5, Cas6, and Cas8*b* results in some amount of crRNAs (32%, Table 3). Thus, the crRNA is generated, but the amount of crRNA is not as high as it is in wild type strains. This result might suggest that the missing Cas7 protein is required for efficient stabilization of the crRNA. In addition, this observation suggests that Cas8b, the large subunit of the type I-B system (59), can protect the crRNA to some extent. The large subunit of the *E. coli* type I-E system (Cse1) was shown to be located close to the crRNA 5′-end, interacting with the PAM sequence of the target DNA (60, 61). Further, it was suggested that Cse1 interacts with two bases in the 5′-handle of the crRNA (62). Thus, the *Haloferax* Cas8b might protect the crRNA from degradation by binding to its 5′-end. Taken together, this suggests that the large subunits of the I-B and the I-E system have similar roles in binding the crRNA and PAM recognition.

A comparison of the Cascade complexes from the different type I systems (IA (22), IE (58, 63, 64), and IF (24, 63)) shows that they all contain multiple copies of the Cas7 protein (in type I-F, this is probably the Csy3 (59, 63)) and that the Cas7 protein forms the backbone of the complex (14). In addition, type I-A and I-E complexes contain the Cas5 protein that interacts with Cas7. The Cas6 protein is also associated with the type I systems either as an integral part (I-E and I-F) or transiently (I-A) (14). Furthermore, these complexes can contain a small subunit (Csa5 in I-A and Cse2 in I-E) and/or a large subunit (Cas8 in type I-A, Cse1 in type I-E, and Csy1 in type I-F) (14). The data presented here for the *Haloferax* type I-B Cascade complex fit very well to these general observations for the type I Cascade complexes. Cas5, Cas6, and Cas7 co-purify, showing that the *Haloferax* complex consists of these subunits. Furthermore, the three Cas proteins are present in almost the same ratio as in the *E. coli* Cascade complex. The crRNA obviously needs Cas5–7 proteins to be generated and to be stably maintained. Structural analysis will show whether the Cas7 protein likewise forms the backbone of the complex binding and protecting the crRNA and whether the Cas8b protein represents the large subunit of the complex.

In addition, these data clearly show that the Cas1–4 proteins are not involved in this reaction. This is in contrast to the results observed with the type I-B system in *H. mediterranei*, in which the deletion of the *cas1*, *cas3*, and *cas4* genes resulted in lower levels of crRNA (12).

## Supplementary Material

Supplemental Data

## Footnotes

3 K. Sharma, B. Stoll, F. Pfeiffer, A. Marchfelder, and H. Urlaub, manuscript in preparation.

## Footnotes

2 The abbreviations used are:

CRISPR: clustered regularly interspaced short palindromic repeats
Cas: CRISPR-associated
crRNA: CRISPR-derived RNA
Cascade: CRISPR-associated complex for antiviral defense
iBAQ: intensity-based absolute quantification.
