# Supplementary material for: A Complex of Cas Proteins 5, 6, and 7 Is Required for the Biogenesis and Stability of Clustered Regularly Interspaced Short Palindromic Repeats (CRISPR)-derived RNAs (crRNAs) in Haloferax volcanii
Source: J Biol Chem. 2014 Jan 23;289(10):7164–77. doi: 10.1074/jbc.M113.508184 (PMC3945376; doi:10.1074/jbc.M113.508184)
Supplement: Supplemental Data [file supp_289_10_7164__index.html]

A complex of Cas proteins 5, 6, and 7 is required for the biogenesis and stability of crRNAs in Haloferax volcanii — A Complex of Cas Proteins 5, 6, and 7 Is Required for the Biogenesis and Stability of Clustered Regularly Interspaced Short Palindromic Repeats (CRISPR)-derived RNAs (crRNAs) in Haloferax volcanii — crRNA Biogenesis and Maintenance in Haloferax — Supplemental Data 

# A Complex of Cas Proteins 5, 6, and 7 Is Required for the Biogenesis and Stability of Clustered Regularly Interspaced Short Palindromic Repeats (CRISPR)-derived RNAs (crRNAs) in *Haloferax volcanii*

## Supplemental Data

**Files in this Data Supplement:**

- SupplementalData (.pdf, 203 KB) - SupplementalData
