# Supplementary material for: A Complex of Cas Proteins 5, 6, and 7 Is Required for the Biogenesis and Stability of Clustered Regularly Interspaced Short Palindromic Repeats (CRISPR)-derived RNAs (crRNAs) in Haloferax volcanii
Source: J Biol Chem. 2014 Jan 23;289(10):7164–77. doi: 10.1074/jbc.M113.508184 (PMC3945376; doi:10.1074/jbc.M113.508184)

Supplementary Table 1. Primers used in this study.

Name	Sequence (5’ >3’)
6iY15Af	[Phos]ATGCGGCCGCCGACCGG
6iY15Ar	[Phos]CAGCAACGGCATCGAGCGCT
6iY19Af	[Phos]ACCGGTCTGCCTTCCATAAGG
6iY19Ar	[Phos]CGTAGGCCGCATCAGCAACG
6iK22Af	[Phos]TCTTATTTCCATGCGGCACGCG
6iK22Ar	[Phos]CCGGTCGTAGGCCGCATCA
6iR26Af	[Phos]ACGCGGAGCCATCTGGCG
6iR26Ar	[Phos]GCCTTATGGAAATAAGACCGGTC
H21A	ACGACCGGTCTTATTTCGCTAAGGCACGCGGACGC
H21Ar	GCGTCCGCGTGCCTTAGCGAAATAAGACCGGTCGT
iW28A	[Phos]ACGCATCGCGCGCGGCCTCGAGAA
iW28Ar	[Phos]CCGCGTGCCTTATGGAAATAAGACCGG
H41A	ACGTTCTCGGAGACCGCCGATTCGAACCACGGCGTC
H45A	ACCCACGATTCGAACGCCGGCGTCGGGTTTTC
H45Ar	GAAAACCCGACGCCGGCGTTCGAATCGTGGGT
H41Ar	GACGCCGTGGTTCGAATCGGCGGTCTCCGAGAACGT
F49A	AACCACGGCGTCGGGGCTTCGTACTCTAATATC
F49Ar	GATATTAGAGTACGAAGCCCCGACGCCGTGGTT
Y51A	ACGGCGTCGGGTTTTCGGCCTCTAATATCTTCCCTTG
Y51Ar	CAAGGGAAGATATTAGAGGCCGAAAACCCGACGCCGT
6iR66Af	[Phos]GACCGGGCCTACTTCCGCA
6iR66Ar	[Phos]GCCCTCTTCTATCTGTCCCCA
S115A	ACGTGGGTGAGGCCGGAGCGACCGGTGTCCTCGAGACG
S115Ar	CGTCTCGAGGACACCGGTCGCTCCGGCCTCACCCACGT
T116A	TGGGTGAGGCCGGATCGGCCGGTGTCCTCGAGACG
T116Ar	CGTCTCGAGGACACCGGCCGATCCGGCCTCACCCA
L172A	ATCCGGCGGTCGGCCCAACAGACACACG
L172Ar	CGTGTGTCTGTTGGGCCGACCGCCGGAT
6S224Af	CACCGTTCTGCTTGCGAAGTGGCGCTTC
6S224Ar	GAAGCGCCACTTCGCAAGCAGAACGGTG
W226A	TTCTGCTTTCGAAGGCGCGCTTCGGCTAC
W226Ar	GTAGCCGAAGCGCGCCTTCGAAAGCAGAA
G248A	TCGCGCTCGATTCGGCGATTGGACAGCGAC
G248Ar	GTCGCTGTCCAATCGCCGAATCGAGCGCGA
G250A	TCGATTCGGGGATTGCACAGCGACGCGAAC
G250Ar	GTTCGCGTCGCTGTGCAATCCCCGAATCGA
G256A	AGCGACGCGAACACGCGTTTGGCTTCCTGAAC
G256Ar	GTTCAGGAAGCCAAACGCGTGTTCGCGTCGCT
F257A	AGCGACGCGAACACGGGGCTGGCTTCCTGAACCTC
F257Ar	GAGGTTCAGGAAGCCAGCCCCGTGTTCGCGTCGCT

G258A	ACGCGAACACGGGTTTGCCTTCCTGAACCTCAC
G258Ar	GTGAGGTTCAGGAAGGCAAACCCGTGTTCGCGT
FLAGN	CCCTAATATATCATATGGACTACAAAGACCATGACGG
FLAG2	TAATATAAGCTTTTTATCGTCGTCATCTTTGTAG
Cas6FlagNup	TAATATATAAGCTTGTGCGTATAGAATTAGCGCTCGATGC
Cas6FlagNdo	TAATATATGAATTCTCAGTCACTCGCCCGTGGAAGCGTTTTG
Cas6FlagCup	TAATATATCATATGGTGCGTATAGAATTAGCGCTCGATGC
3-Cas5-BamHI	TAATATGGATCCTTAGCGGAAGACGACGGTCCGGTC
3-Csh2-HindIII	ATATATAAGCTTTTAGGCGAGGTCGCGTTCGTTGAGC
5-Cas6-NcoI	TTATAACCATGGCTGGCGTGCGTATAGAATTAGCGCTC
3-Cas6-NotI	TTATAAGCGGCCGCTCAGTCACTCGCCCGTGGAAGCGTTTT
3-Cas6-HindIII	TATTATAAGCTTGTCACTCGCCCGTGGAAGCGTTTTG
5-Cas6-PciI	TTATAAACATGTCGGGCGTGCGTATAGAATTAGCGCTC
Cas6KOup	CCTTGTGGGATTGAAGCCGGCTCG
Cas6KOdo	AGCCGGAGGCGGACTGTGATGACG
IPCas6KOup	[Phos]GCCTCATATCACCCCTCTCAAAGTTGTG
IPCas6KOdo2	ATGACAGGTCCAGATATCGACGACTTCG
Csh1#2	CCTTTCTCGAAGACCGCCGCGAGCTC
Csh2#2	CTCGACGCGGACGTAGAGCCGCGGC
Cas5#2	CCTCGATAACCGCTAAGTACTCCGAC
Cas3#5	GACGTGTGATTCGGGAACTGCCATCG
Cas4#2	CCAACAGAGGTCTTGGTACAGACAG
Cas1#5	GAGTGCATTCGTTTCGTTACTCGGTGG
Cas2#2	GGTTGTCGAATTCGAAGACGACAATC
Cas6#4	GCGTTCGAGACGAGACACACCGATACCAC
Csh1#3	GAGCTCCAGTTCTGCTACGTGTTAGGC
Csh2#3	GACACGCTCCACGTGGTTGGAGATGGC
Cas5#3	GATGACTACGTCTACGCACAGCAAGC
Cas4#4	CGGAGTACTAGACGTGGTGGGCAGG
Cas1#4	GCTTCAAGACGCTCATTCAGACGGAC
Cas6#3	CGACGTCGTCTTTGATCGGCTCGATG
Cas6#2	CGGTTCCATGCCGTGCTTCGGACGC
Mmarfwdcas6	TAATATCATATGGATTTAGAATACATGC
Mmarrevcas6	GAACTGTAGTAAAAGTTTAAGAATTCTATATA
P1.1	GTTCCGGGAGGTCGCCGGTCGAGATGCCTGC
P1.2	GGTTGCGTGTAACCGTCTTCTTTGATGGACTTG
P2.1	TATTGCCTATGCCCGGCAGAACGTCCACCCCGA
C1	CTGAGATTCGAGGGCATCTTCGGACCTTTCC
5S	CGCAGGTGAGCTTAACTTCCGTGTTCGGG
hdrB-dNsp F	GGTGCCACGGCATaTCGCCGTCGCGG
hdrB-dNsp R	CCGCGACGGCGAtATGCCGTGGCACC
FlagSnaBI	TAATATTACGTAGACTACAAAGACCATGACGG
HisFlagBamHI	TAATATGGATCCATGCATCACCACCACCATCACGTAGAC

3-Cas5-XbaI	TAATATTCTAGATTAGCGGAAGACGACGGTCCGGTC
3-Cas8-NcoIBamHI	TAATATGGATCCCCATGGTTAGTTCGTGGTGCTCTCAGC

Supplementary Table 2. Strains and plasmids used in this study.

**Strains**	**Genotype**	**Source/Reference**
DH5α	F^- φ80*lac*ZΔM15 Δ(*lac*ZYA-*arg*F) U169 *rec*A1 *end*A1 *hsd*R17 (rk-, mk+) *gal*- *pho*A *sup*E44 λ- *thi*-1 *gyr*A96 *rel*A1	Life technologies
GM121	F^- *dam-3 dcm-6 ara-14 fhuA31 galK2 galT22 hdsR3 lacY1 leu-6 thi-1 thr-1 tsx-78*	Allers *et al.,* 2010
BL21AI	F^- *omp*T *hsd*S_B(r_B^- m_B^-) *gal dcm araB:T7RNAP-tetA*	Life technologies
H119	Δ*pyrE2* Δ*trpA* Δ*leuB*	Allers *et al.,* (2004)
H119Δ*cas7*	Δ*pyrE2,* Δ*trpA,* Δ*leuB,* Δ*cas7*	This study
H119Δ*cas8*	Δ*pyrE2,* Δ*trpA,* Δ*leuB,* Δ*cas8*	This study
H119Δ*cas6*	Δ*pyrE2,* Δ*trpA,* Δ*leuB,* Δ*cas6*	This study
H26*casCluster28*	Δ*pyrE2*, Δ*cas* (ΔpHV4: 207.288-218.340)	Fischer *et al.* 2012
H1424	Δ*pyrE2* Δ*hdrB* $pitA_{Nph}$ Δ*mrr*, *cdc48d* c-terminal truncation	Straud et al., 2012
**Plasmids**	**Relevant properties**	**Source/Reference**
pTA927-*cas6*	Shuttle vector with *pyrE2*-marker, pHV2 replication origin, P.*tnaA* tryptophanase promoter, T.syn terminator, n-terminal 3XFLAG-tag and *cas6* gene from *Haloferax volcanii*	This study
pTA927-Mmaz*cas6*	Shuttle vector with *pyrE2*-marker, pHV2 replication origin, P.*tnaA* tryptophanase promoter, T.syn terminator, *cas6* gene from *Methanosarcina mazei*	This study
pTA927-Mmar*cas6*	Shuttle vector with *pyrE2*-marker, pHV2 replication origin, P.*tnaA* tryptophanase promoter, T.syn terminator, *cas6* gene from *Methanococcus maripaludis*	This study
pTA1228	Shuttle vector with *pyrE2*-marker, pHV2 replication origin, P.*tnaA* tryptophanase promoter, T.syn terminator and n-terminal 6XHis-tag	This study
pTA1228-Hlac3333	Shuttle vector with *pyrE2*-marker, pHV2 replication origin, P.*tnaA* tryptophanase promoter, T.syn terminator, n-terminal 6XHis-tag and *cas6* gene Hlac3333 from *Halorubrum lacusprofundi*	This study
pTA1228-Hlac3572	Shuttle vector with *pyrE2*-marker, pHV2 replication origin, P.*tnaA* tryptophanase promoter, T.syn terminator, n-terminal 6XHis-tag and *cas6* gene Hlac3572 from *Halorubrum lacusprofundi*	This study
pTA963	Shuttle vector with *pyrE2*-marker, pHV2 replication origin, P.*tnaA* tryptophanase promoter, T.syn terminator	Allers *et al.,* 2010
pTA927-*cas6*mutX	Shuttle vector with *pyrE2*-marker, pHV2 replication origin, P.*tnaA* tryptophanase promoter, T.syn	This study

	terminator, n-terminal 3XFLAG-tag and mutated *cas6* gene containing the different mutated *cas6* genes	
pTA927-*cas6875*<br>pTA927-*cas687*<br>pTA927-*cas685*<br>pTA927-*cas675*	Shuttle vector with *pyrE2*-marker, pHV2 replication origin, P.*tnaA* tryptophanase promoter, T.syn terminator, respective set of *cas* genes	This study
pTA131*cas6*updo	Integrative vector with *pyrE2*-marker, regions upstream and downstream of *cas6* gene	This study
pTA963-*cas6*	Shuttle vector with *pyrE2*-marker, pHV2 replication origin, P.*tnaA* tryptophanase promoter, T.syn terminator, n-terminal 6XHis-tag and *cas6* gene from *Haloferax volcanii*	This study
pET28a-*cas6*	*E.coli* expression vector (Novagen), N-terminal 6XHis-tag and *Haloferax cas6* gene	This study
pET32a-*cas6*	*E.coli* expression vector (Novagen), N-terminal 6XHis-tag, S-tag and Trx-tag, *Haloferax cas6* gene	This study
pET30a-*cas6*-FlagN	*E.coli* expression vector (Novagen), N-terminal 3XFLAG-tag and *Haloferax cas6* gene	This study
pET30a-*cas6*o.stop	*E.coli* expression vector (Novagen), N-terminal 6XHis-and S-tag, C-terminal 6XHis-tag, *Haloferax cas6* gene	This study
pET28a-*cas6*h	*E.coli* expression vector (Novagen), N-terminal 6XHis-tag an *Haloferax cas6* gene (harmonized codon usage)	This study
pET30a-*cas6*h	*E.coli* expression vector (Novagen), N-terminal 6XHis- and S-tag, *Haloferax cas6* gene (harmonized codon usage)	This study
pTA352	Shuttle vector with *leu*B marker and pHV1 replication origin	Norais *et al*., 2007
pTA352-PAM3	Spacer P1.1. downstream of PAM3 (TTC)	Fischer *et al.* 2012
pTA352-PAM9	Spacer P1.1. downstream of PAM9 (ACT)	Fischer *et al.* 2012
pTA927-Cas68[HisFlag]75	Shuttle vector with *pyrE2*-marker, pHV2 replication origin, P.*tnaA* tryptophanase promoter, T.syn terminator, *cas6, cas8, cas7* and *cas5* genes from *Haloferax volcanii*, *cas7* with N-terminal 6xHis and 3XFAG-tag	This study

Supplementary Table 3

iBAQ quantitative mass spectrometry analysis of Cas7 co-purification. Proteins listed were confidently identified and quantified in 3/3 replicate analyses. For the UPS2 standard proteins, nominal amounts in 1 µg standard were used to derive a calibration curve (double logarithmic plot (log(iBAQ) vs. log(amount), Suppl. Fig. x). iBAQ values are averaged from three replicates. Cas5-7 concentrations were calculated using the log/log linear regression y = 0,8932x + 1,3344.

Protein	Source	Protein amount [fmol]	iBAQ	IBAQ StdDev	log (iBAQ)	log (amount)	Ratio
>P12081ups|SYHC_HUMAN	UPS2 Standard	47	426	13	1,674	2,630	
>P16083ups|NQO2_HUMAN	UPS2 Standard	47	457	83	1,674	2,660	
>P61626ups|LYSC_HUMAN	UPS2 Standard	47	858	85	1,674	2,934	
>P06732ups|KCRM_HUMAN	UPS2 Standard	47	862	133	1,674	2,936	
>P02753ups|RETBP_HUMAN	UPS2 Standard	47	617	275	1,674	2,790	
>P63165ups|SUMO1_HUMAN	UPS2 Standard	472	4074	254	2,674	3,610	
>P02144ups|MYG_HUMAN	UPS2 Standard	472	3745	345	2,674	3,573	
>P15559ups|NQO1_HUMAN	UPS2 Standard	472	4791	558	2,674	3,680	
>P01133ups|EGF_HUMAN	UPS2 Standard	472	6039	395	2,674	3,781	
>P62937ups|PPIA_HUMAN	UPS2 Standard	472	6963	854	2,674	3,843	
>Q06830ups|PRDX1_HUMAN	UPS2 Standard	472	6475	170	2,674	3,811	
>P04040ups|CATA_HUMAN	UPS2 Standard	472	6633	463	2,674	3,822	
>P00167ups|CYB5_HUMAN	UPS2 Standard	472	11428	1643	2,674	4,058	
>P69905ups|HBA_HUMAN	UPS2 Standard	4717	22246	1469	3,674	4,347	
>P68871ups|HBB_HUMAN	UPS2 Standard	4717	27773	4132	3,674	4,444	
>P62988ups|UBIQ_HUMAN	UPS2 Standard	4717	33625	3406	3,674	4,527	
>P00918ups|CAH2_HUMAN	UPS2 Standard	4717	40906	6958	3,674	4,612	
>P41159ups|LEP_HUMAN	UPS2 Standard	4717	52631	1567	3,674	4,721	
>P00915ups|CAH1_HUMAN	UPS2 Standard	4717	54385	6349	3,674	4,735	
>P01031ups|CO5_HUMAN	UPS2 Standard	4717	53181	5761	3,674	4,726	
>tr|D4GQN4|D4GQN4_HALVD (**Cas6**)	Co-Purification	249	1259	353	2,396	3,100	**1,0**
>tr|D4GQN7|D4GQN7_HALVD (**Cas5**)	Co- Purification	419	2004	228	2,622	3,302	**1,7**
>tr|D4GQN6|D4GQN6_HALVD (**Cas7**)	Co- Purification	2119	8522	362	3,326	3,931	**8,5**

Supplementary Figure 1
iBAQ Calibration Curve curve (double logarithmic plot (log(iBAQ) vs. log(amount) using 20/48 UPS2 standard proteins that were observed in 3/3 replicate analyses.

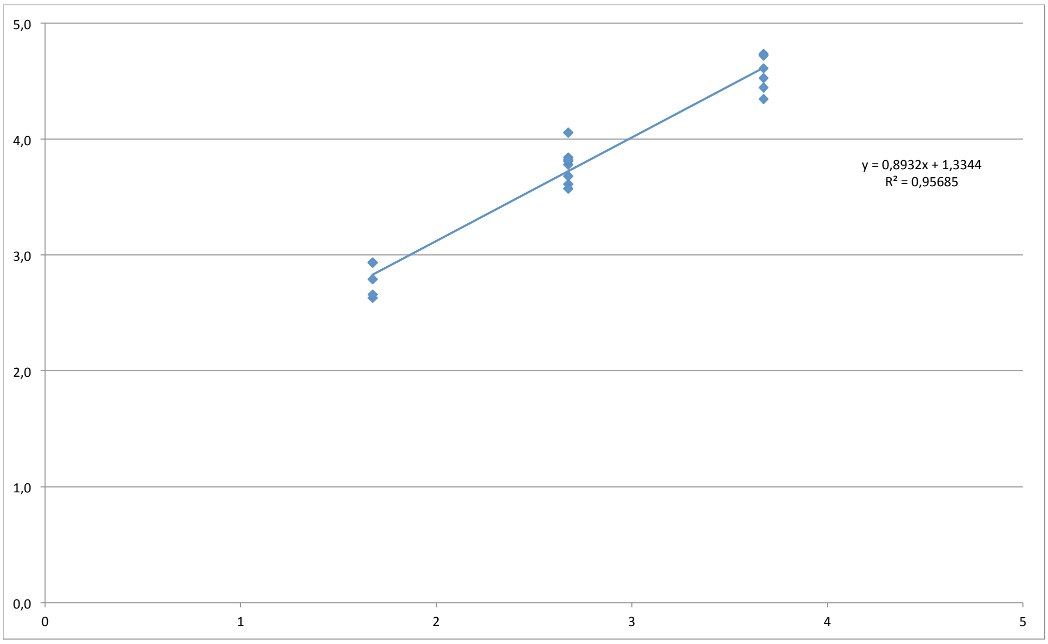

Supplement: Supplemental Data [file supp_M113.508184_jbc.M113.508184-1.pdf]
